# A.S.S.E.S.S. for Facial Fillers

**DOI:** 10.1111/jocd.16633

**Published:** 2024-10-27

**Authors:** John P. Fezza, Sheila Barbarino, Julie Woodward, Reed Fezza, Jonathan D. Tijerina, Wendy Lee

**Affiliations:** ^1^ Center for Sight Sarasota Florida USA; ^2^ Barbarino Surgical Arts Austin Texas USA; ^3^ Duke University Eye Center Durham North Carolina USA; ^4^ Bascom Palmer Eye Institute University of Miami Miller School of Medicine Miami Florida USA

**Keywords:** aging face, dermal filler, facial assessment, facial profile, facial shape, filler, neuromodulation

## Abstract

**Background:**

An in‐depth and detailed facial assessment is critical in treating and achieving desirable dermal filler and neurotoxin results.

**Methods:**

An acronym called A.S.S.E.S.S. simplifies an often complex and overwhelming amount of data needed to assimilate when performing facial filler and neurotoxin injections. Applying this method to patients in six simple steps provides a starting point and offers a guideline to capture key details for a more comprehensive facial assessment. The A.S.S.E.S.S. acronym stands for animate, shape, side, external, symmetry, and shadows and is helpful in following a methodical approach in analyzing facial shape, profile, and natural facial curves in both static and dynamic states.

**Results:**

Following a regimented A.S.S.E.S.S. approach prior to treating filler and neurotoxin patients allows providers a straightforward guide to achieve a desirable facial shape and profile.

**Conclusion:**

This stepwise facial assessment establishes a logical and detailed approach to ensure the important aspects of facial details are appreciated in creating reliable and pleasing filler and neurotoxin results.

## 1 Introduction

Patient assessment for facial fillers and neurotoxins is crucial to deliver an effective and safe injection result. Critical analysis before filler injections is the first step in restoring a patient's natural and youthful appearance. The adage measure three times and cut once applies to the importance of a logical prefiller workup. The neoclassical canons still resonate in describing the ideal facial proportions, and they can be useful in planning nonsurgical facial rejuvenation with fillers [1]. Prior published descriptions on filler assessment have been sparse but include the use of the golden rule or phi ratio in restoring a youthful appearance with fillers [2]. Other articles on facial assessments for fillers have focused on anatomy, aging, and facial proportions [3, 4].

A reproduceable, methodical sequence of steps allows an injector to follow a logical and predicable approach to facial analysis. The acronym A.S.S.E.S.S. (Animate, Shape, Side, External, Symmetry, and Shadows) is a simple, easy to remember, and repeatable method to evaluate key aspects of a patient's facial form. This manuscript reviews a system for facial assessment for fillers and neurotoxins that allows an injector to capture the important aspects for delivering results. The significance of each letter of the A.S.S.E.S.S. acronym is detailed.

### 1.1 A: Animation

Facial animation is a key aspect of any patient assessment as it relates to dynamic changes in facial shape with movement. We live in dynamic motion and not as statues and failure to realize this can result in unnatural, mask‐like results when performing facial procedures. Having a patient smile, frown, pucker, and raise brows will allow an evaluation of the facial nerve, muscles, and asymmetries. Facial palsy is important to note prior to toxin injections as a patient might erroneously assume the unwanted weakness was the result of the toxin injection. Synkinesis of the facial nerve can also be present in the form of abnormal movements stemming from miswiring of the seventh cranial nerve. One example is a pathologic misdirection of the zygomatic and buccal branches of the facial nerve and manifest as a lip twitching with eyelid closure. Also, cheek elevation is present in a sincere smile, or Duchenne smile, as the cheek elevators are recruited in a natural smile affecting the eyes. Cheeks that are previously over filled with dermal filler can crowd the eyes, making them look small and sunken. This should be a warning sign to avoid placing more filler into the cheek area, or even an opportunity to reverse the large cheek mounds. Educating the patient about any of these concerns prior to filler or toxin injections is important so as not to be blamed for an undesirable outcome.

### 1.2 S: Shape of a Face on Frontal View

**FIGURE 1:**
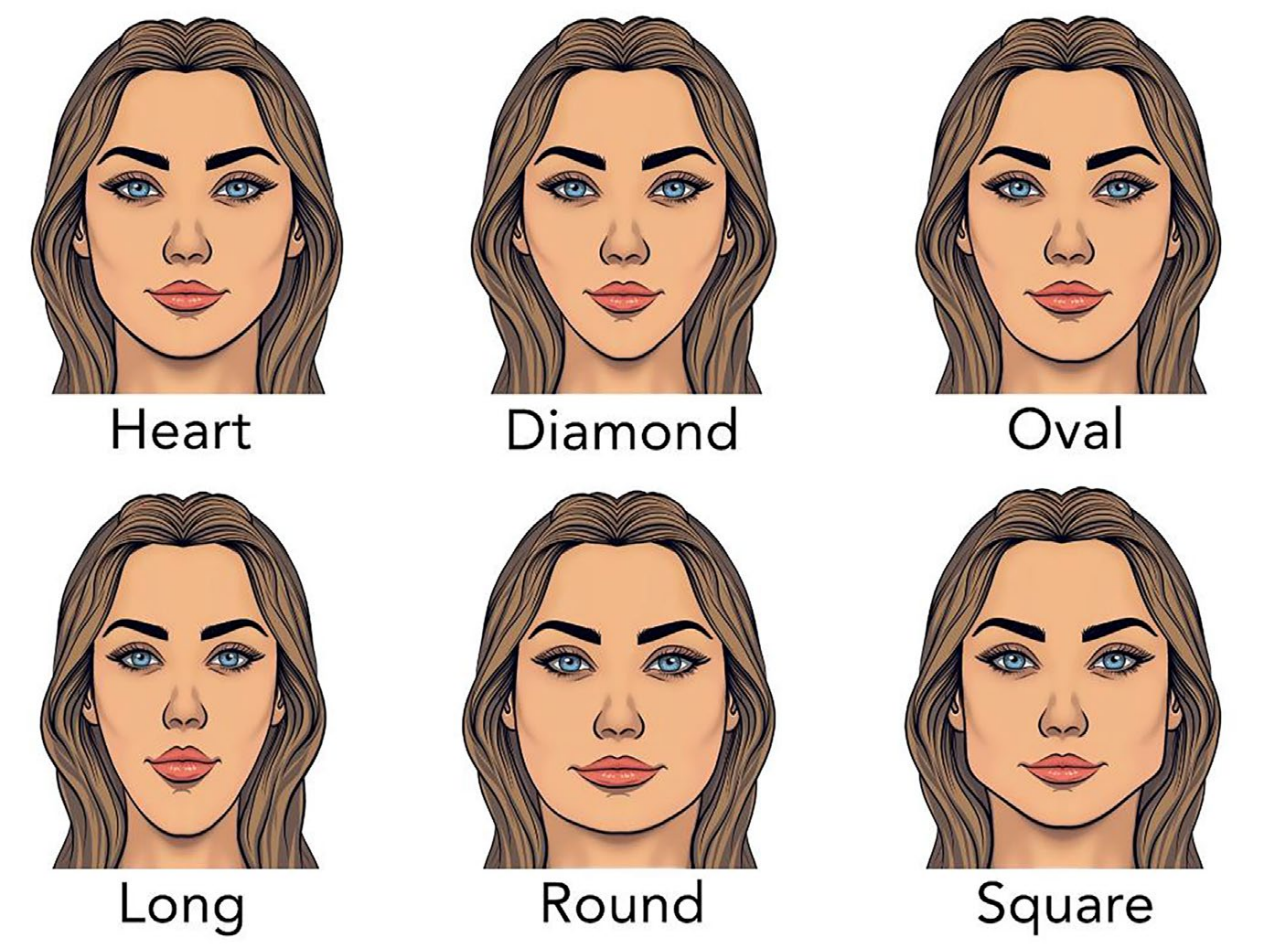
The six basic facial shapes are heart, diamond, oval, oblong, round, and square/rectangle. The heart shape is the most desirable female shape, while the square/rectangle is the most desirable for a male face.

**FIGURE 2:**
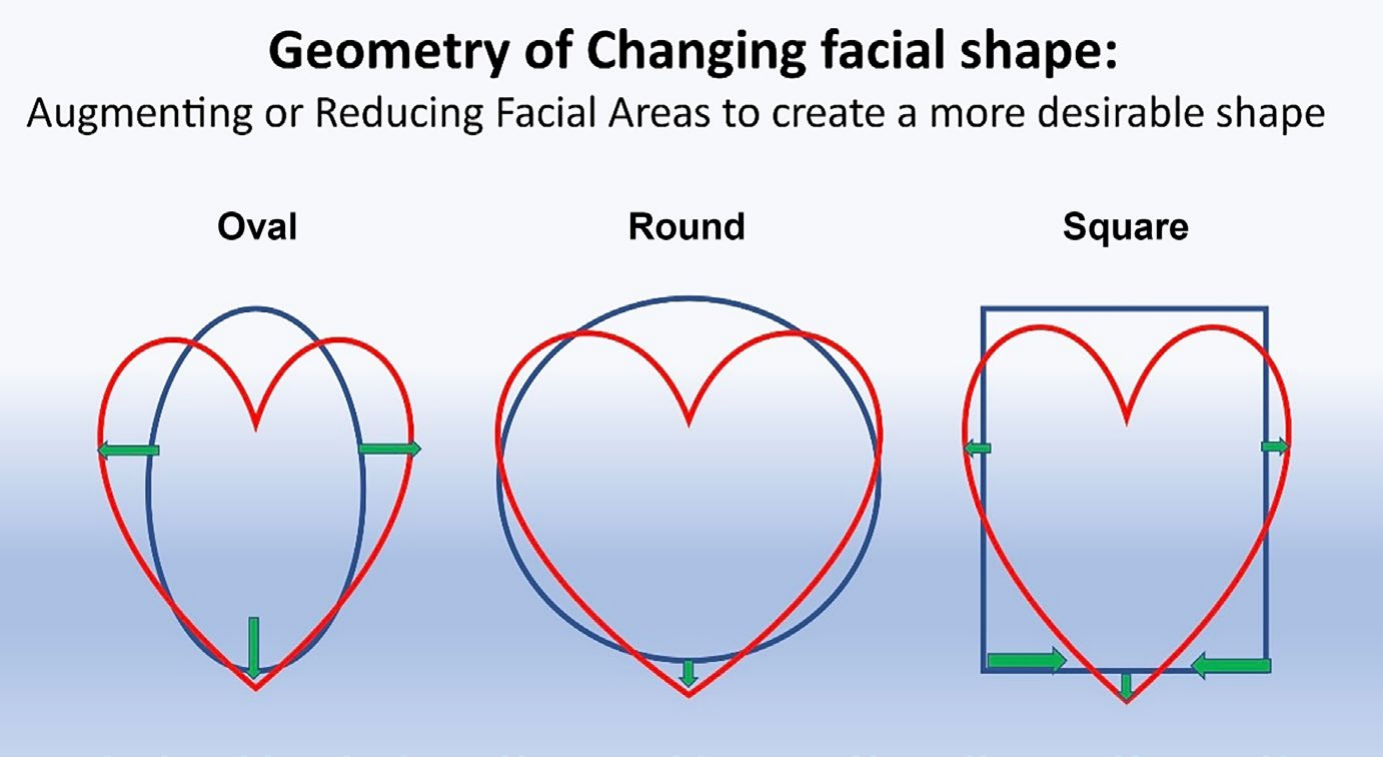
Basic geometric shapes can be modified with filler to transform one shape into a more pleasing one. For example, if a patient has an oval face, then filler can be added to the cheeks and point of the chin to create a more heart shape. Likewise, if a person's face is squarer, then filler can be added to the lateral cheek and central chin, while also slimming the face by reducing lower facial width with injections of neurotoxin to the masseter muscles.

**FIGURE 3:**
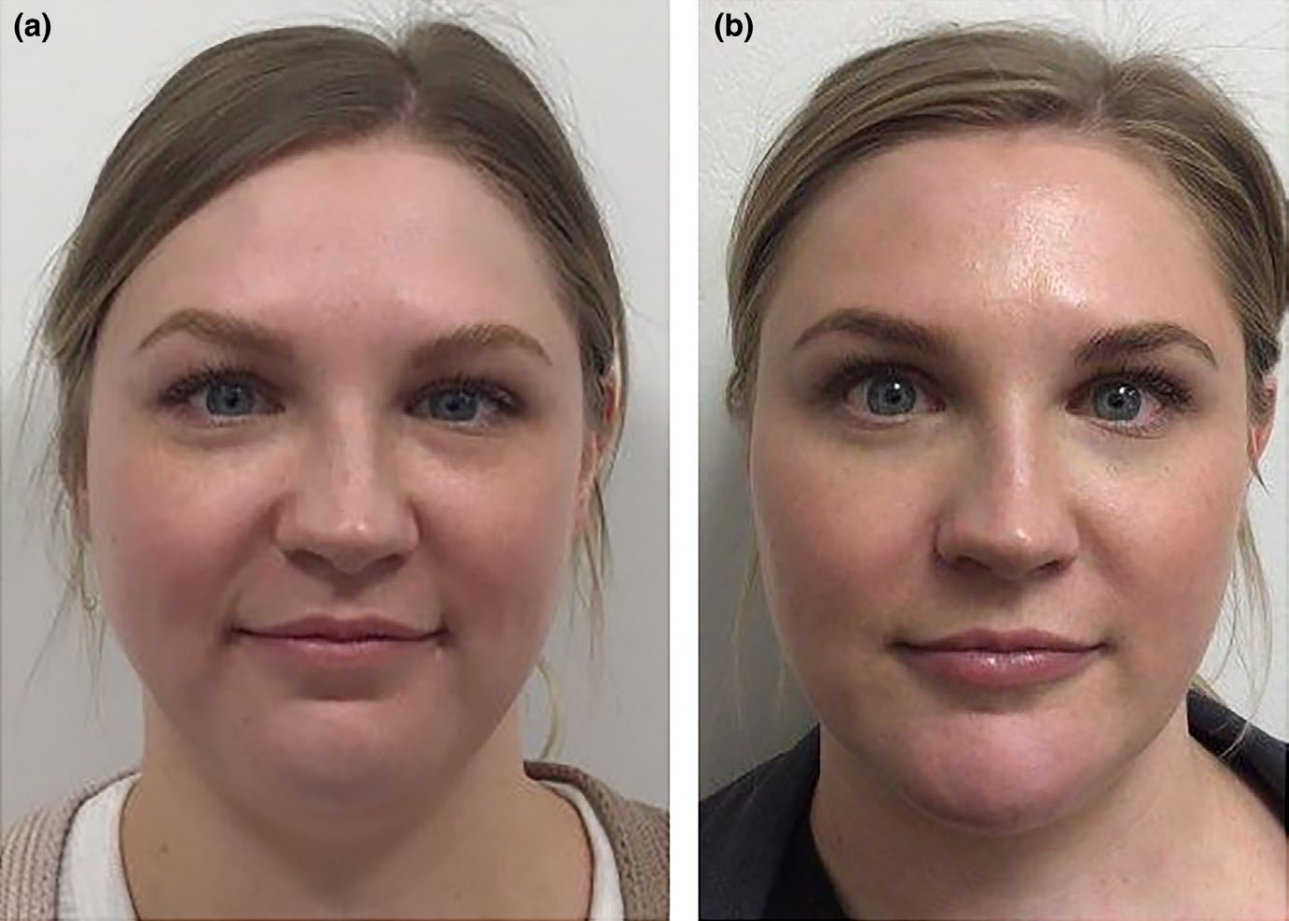
(a) A clinical example of a patient with a round facial shape. A rounder facial shape is often associated with chin deficiency, especially regarding length. (b) Same patient converted from a round to a more heart shape with 8 mL of HA filler. A high G' filler was injected with a cannula in the lateral cheeks and central chin to lengthen her face. A total of 6 mL was used to improve facial shape in addition to 1 mL of HA filler in her tear trough and lips. It is fascinating that adding filler can have the overall effect of facial slimming when placed appropriately (photograph courtesy of Dr. Drew Taylor, Aspen CO).

**FIGURE 4:**
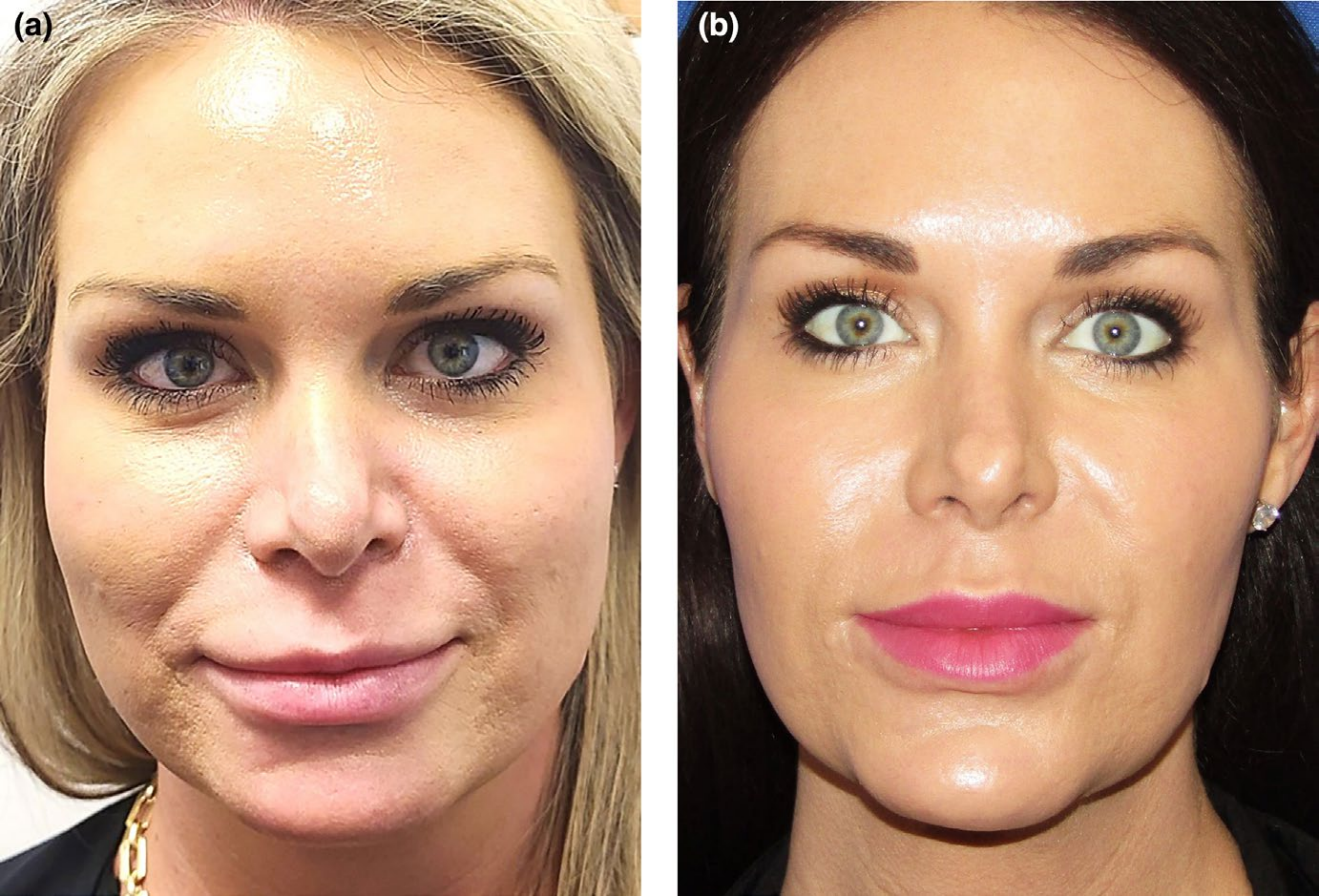
(a) Patient with a square facial shape and strong masseters from teeth grinding. (b) Same patient after 2 mL pf high G' HA filler to lateral cheeks and 1 mL to her chin point with a cannula in subcutaneous plane. Her masseters were injected with 50 units of neurotoxin to slim her lower face. The result is a transformation of a square facial shape into a more heart‐shaped face (photograph courtesy of Dr. John Fezza, Sarasota, FL).

**FIGURE 5:**
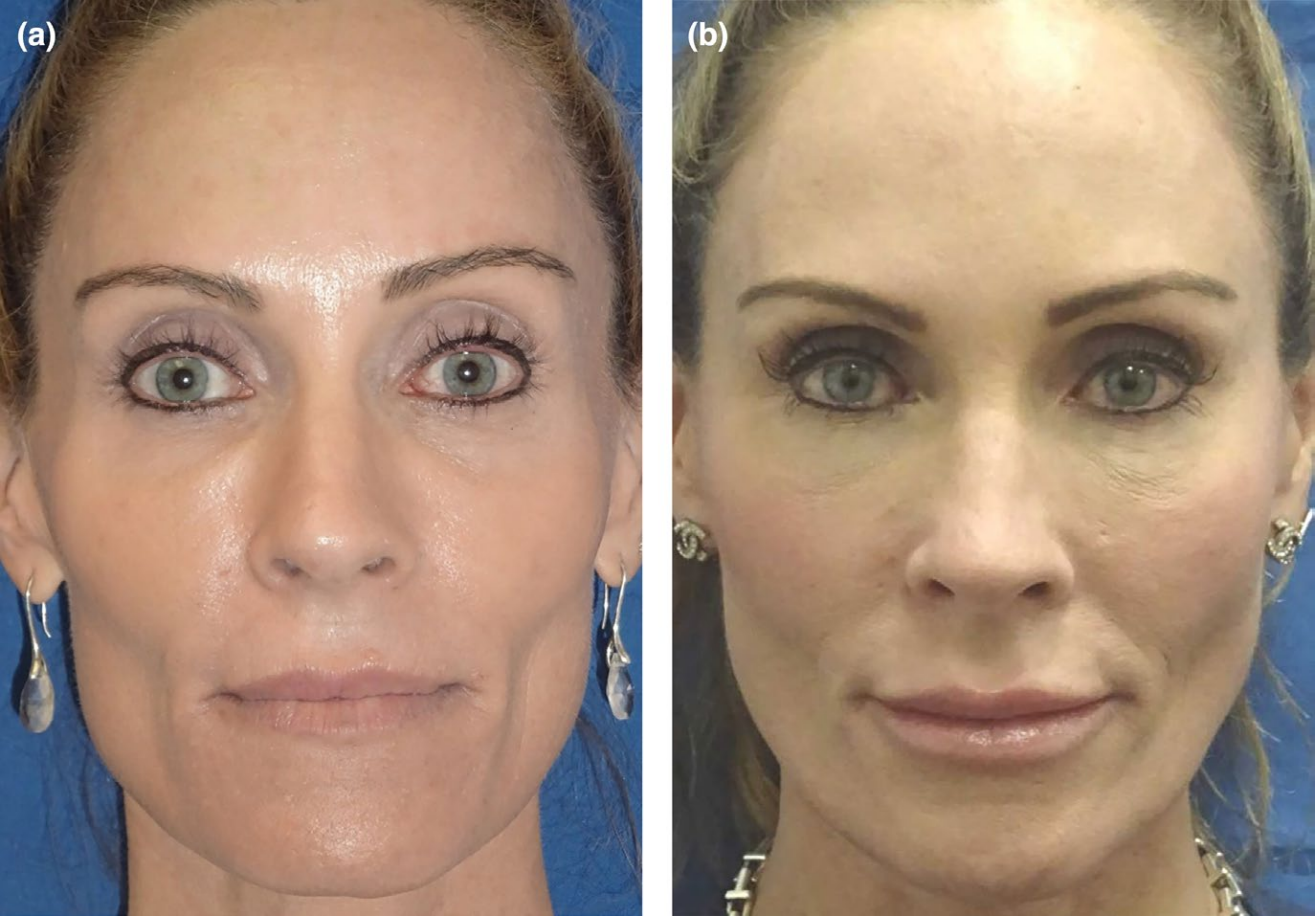
(a) This athletic patient had low body fat and a more rectangular facial shape. (b) She received 3 mL of High G' HA filler to her cheeks with deep, periosteal needle injections, in addition to 1 mL of HA filler with cannula to her tear trough deep to her orbicularis oculi muscle and 1 mL of HA filler with needle to her lips. She also had 40 units of neurotoxin to her masseter muscles to create a tapered lower face and more heart shape (photograph courtesy of Dr. John Fezza, Sarasota, FL).

**FIGURE 6:**
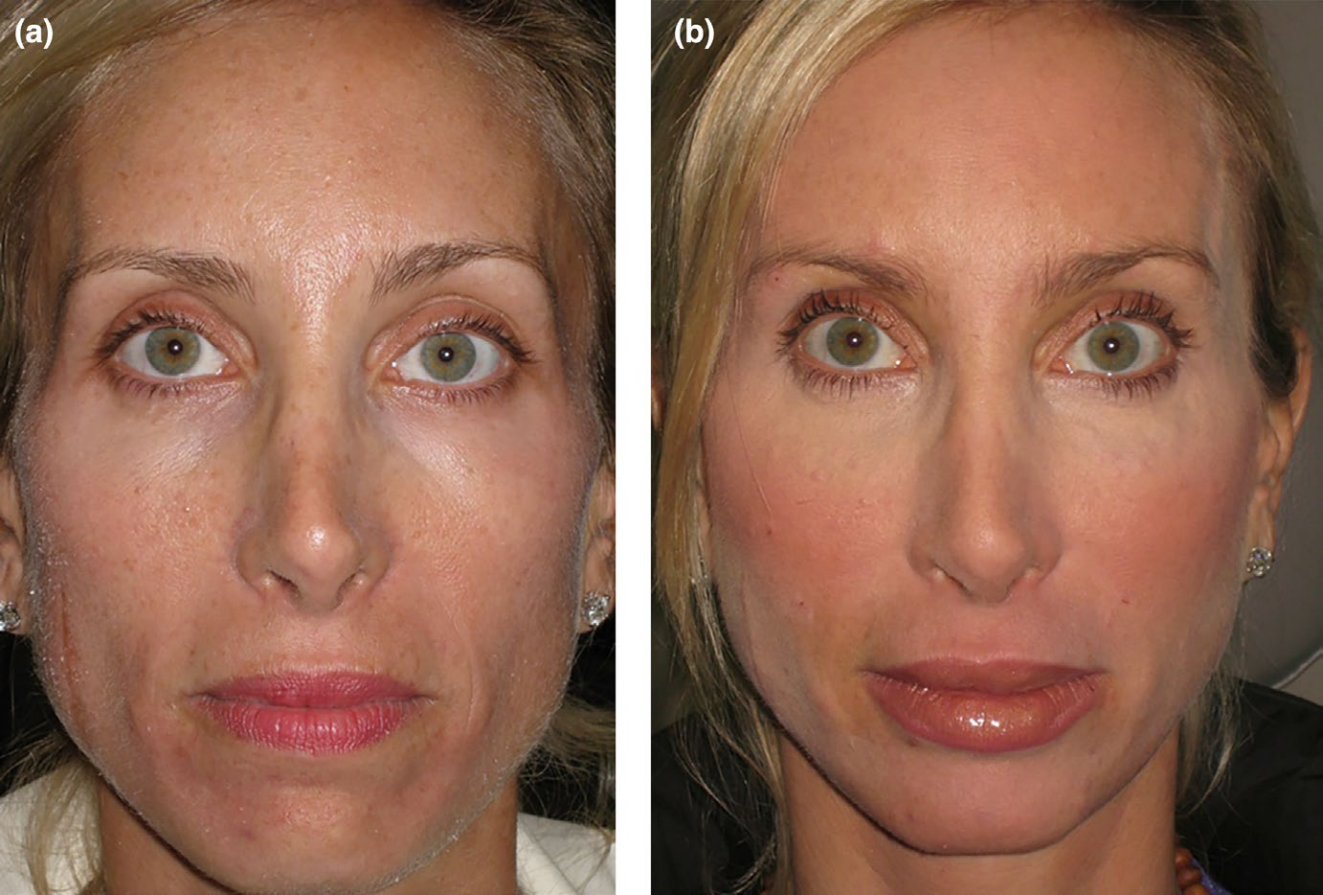
(a) This patient desired upper face rejuvenation and displayed temple hollowing, mid face deflation and hollow tear troughs. (b) Same patient after 2 mL high G' HA deep temple filler delivered with needle on bone, 2 mL of HA mid face filler, and 1 mL of low hydrophilic HA filler with cannula to her tear troughs (photograph courtesy of Dr. Julie Woodward, Durham, NC).

Facial assessment on a front view is important as this is the view a patient sees in the mirror. Analysis of a front view typically reveals one of six basic shapes: oval, round, square, rectangular, heart, and diamond (Figure 1). The heart shape is most desirable for a female, while the rectangular suits a male patient best. The advent of robust hyaluronic acid (HA) filler products has allowed filler injectors to evolve into facial sculptors. Facial shape can be enhanced or modified with these newer lifting structural fillers, so understanding the original facial shape and the desirable shape is important. Drawing the patient's simple geometric shape and then overlying the desired shape, such as a heart, is a straightforward way to show where filler augmentation is most needed, or where volume needs to be reduced (Figure 2). For example, if a female patient has a round face and wants a more heart shape, then overlying a heart over the circle demonstrates more lateral cheek volume and chin projection is desired and filler can be placed in these deficient areas (Figures 3, 4, 5). Conversely if a female patient has a square‐shaped face, then conversion to a more pleasing heart‐shaped face can be achieved with increasing lateral cheek and inferior chin projection in addition to reducing the wider lower half of the face. This can be achieved by adding a dermal filler of high G' to the cheeks and chin, while reducing the masseter width using neurotoxin injections to thin the muscle and slim the lateral lower face. Once basic facial shape has been maximized, more delicate areas such as the tear trough can be addressed secondarily in a more conservative fashion (Figure 6).

**FIGURE 7:**
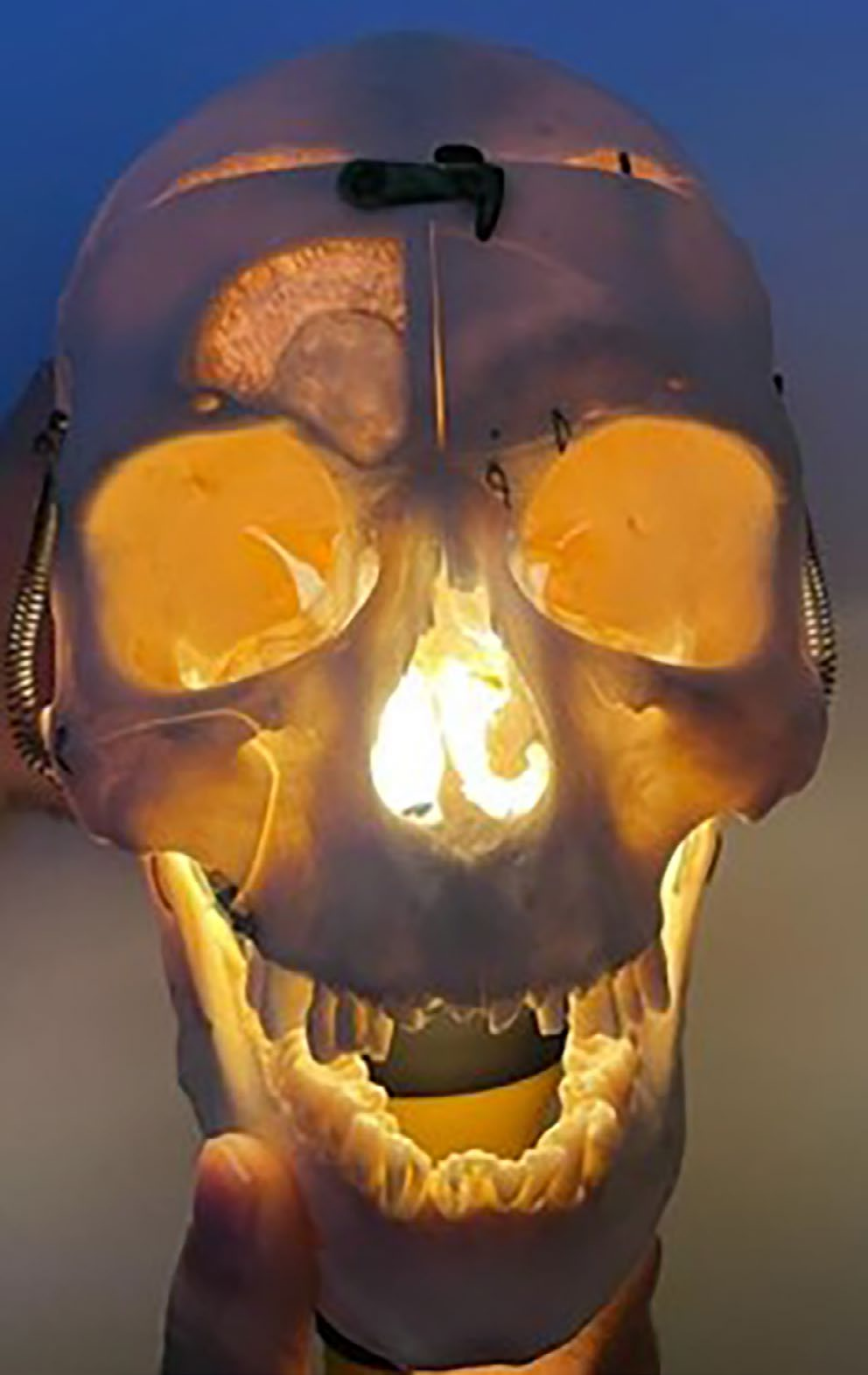
Transillumination of the skull reveals thin bone that transmits light and thick bone that does not. The thicker bones are buttress areas of the skull and are used for stability points when fixating broken facial bones. These same thick buttress areas of the skeleton are convexities and are also the regions filler is best placed to create lift in the face. The lateral brow, cheek, pyriform, chin, and gonial areas contain thicker bone that do not transilluminate and are typically the regions that produce the most projection when filler is injected.

The ability to alter frontal shape is based on the deeper understanding that facial shape is determined by multiple deep underlying anatomic factors such as skeletal framework, fat pad location and prominence, and muscular activity. The skeleton is the foundation of the face and is largely responsible for facial shape. In this regard, volumizing fillers can be beneficial in augmenting bony areas that are depleted or atrophic with age such as temples, cheeks, chin, and jawline. These areas are typically convexities with underlying thick bone. Transilluminating the skull can demonstrate these areas of thick bone, and these regions are targets for deep preperiosteal filler injection as they represent the zones of greatest soft tissue lift over solid bone support (Figure 7). Convexities of the upper and mid face have deep immobile fat overlying them for protection and are called the ROOF (retro‐orbicularis oculi fat) in the brow region and SOOF (suborbicularis oculi fat) in the cheeks. The greatest improvement in surface coefficient or cheek projection has been shown when injecting filler in the SOOF layer, and injectors can use this knowledge to enhance facial structure [5].

**FIGURE 8:**
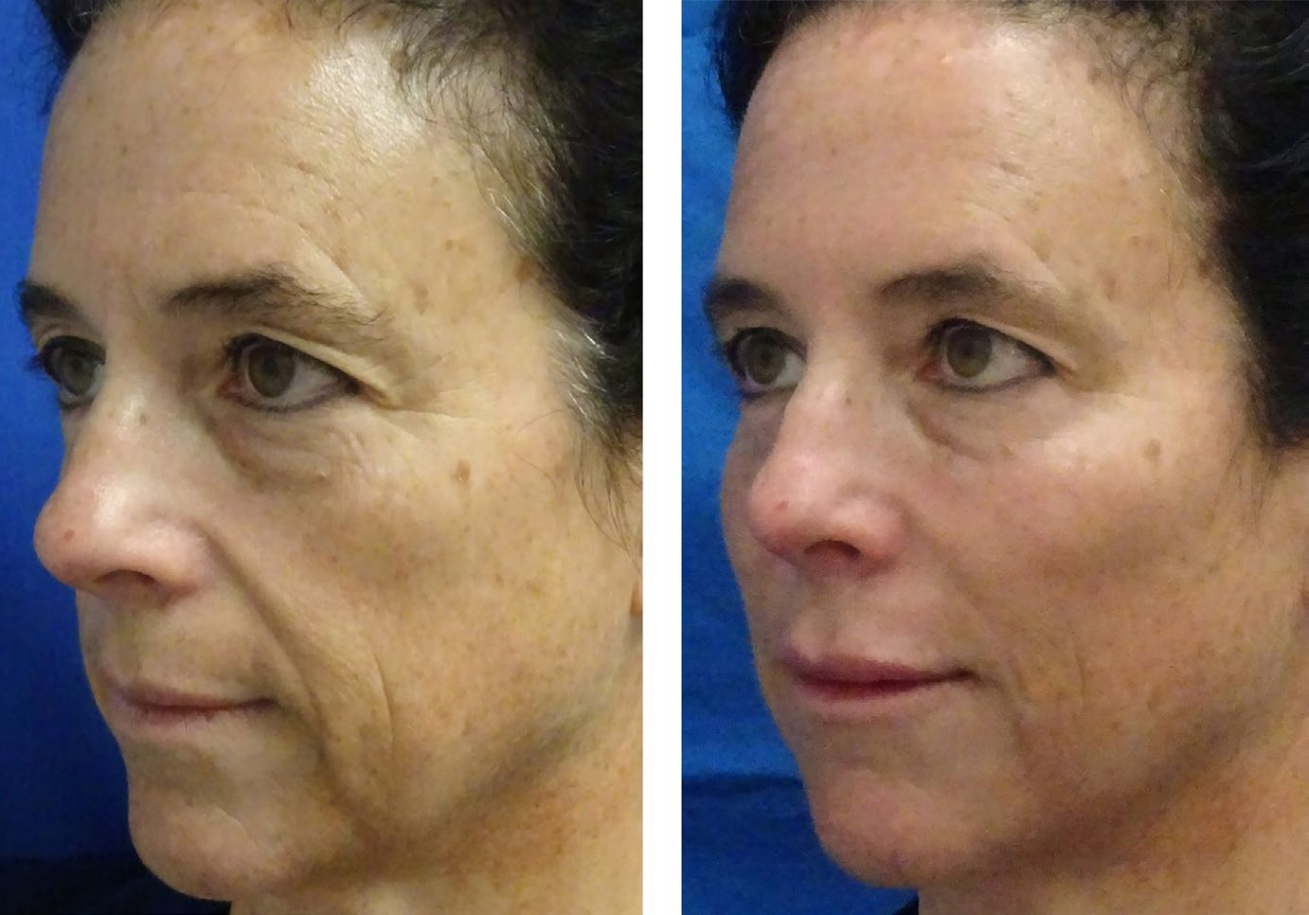
This oblique view demonstrates the pathologic signs of aging with fatty deposition in the superficial nasolabial and jowl fat compartments creating a medial fullness and facial sagging. The loss of fat and facial deflation is evident laterally. Restoring a youthful appearance was achieved by injecting 3 mL of high G' HA filler with cannula primarily in the subcutaneous plane lateral to the line of ligaments in the cheek, which resulted in a filling and lifting effect. 1 mL of filler was also placed in the tear troughs, marionette lines (1 mL) and nasolabial fold (1 mL). A total of 6 mL of filler was injected (photograph courtesy of Dr. John Fezza, Sarasota, FL).

Facial shape on a frontal view is also determined by the muscles of mastication. A vertical line of facial ligaments separates the medial from the lateral face [6]. The medial face contains the facial mimetic muscles, while lateral to the line of ligaments are the muscles of mastication. These muscles of mastication are not responsible for active facial movement but rather are important in determining facial shape. For example, a square facial shape can be slimmed with appropriate use of neurotoxin injections into bulky masseter muscles. Tissue layers lateral to the line of ligaments are arranged in more parallel layers and are responsive to not only volumizing but also lifting with fillers. Studies have verified there is significant fatty soft tissue loss with age in the lateral face, and volumizing this area first can create a lifting effect on the sagging lower face [7] (Figure 8).

#### 1.2.1 S: Side or Sagittal View

**FIGURE 9:**
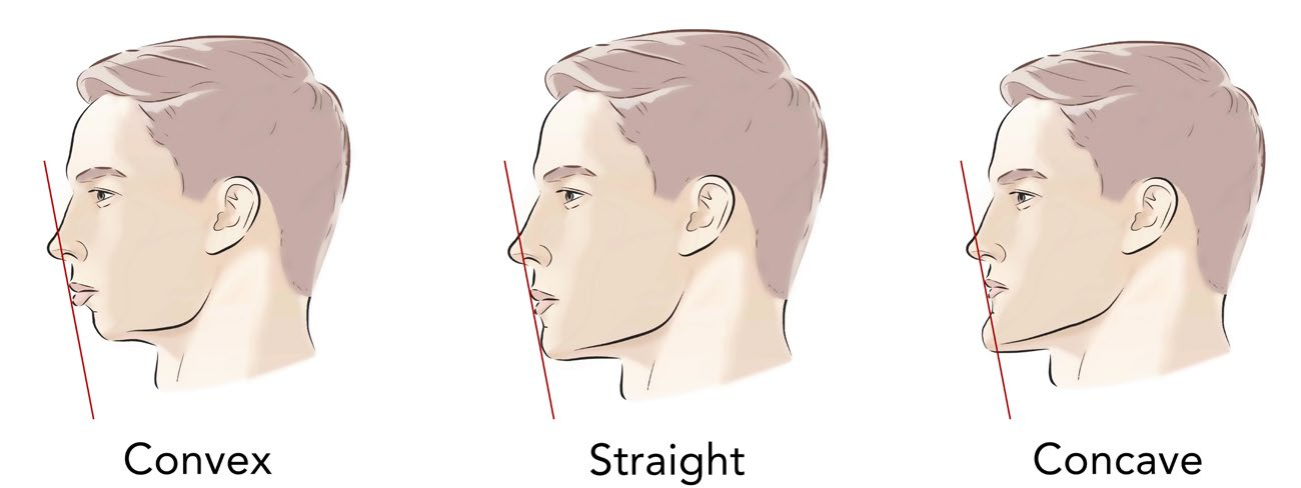
There are three basic facial profiles, convex, straight, and concave. The lower face profile can be assessed with Reidel's Plane, which is a line that connects the most prominent portion of the upper and lower lips and touches the soft tissue menton. If the chin falls behind this line, it means that there is microgenia and a convex profile. The convex lower face is heralded by chin recession and often type 2 dental malocclusion and benefits from adding filler to the chin to create a straighter profile.

**FIGURE 10:**
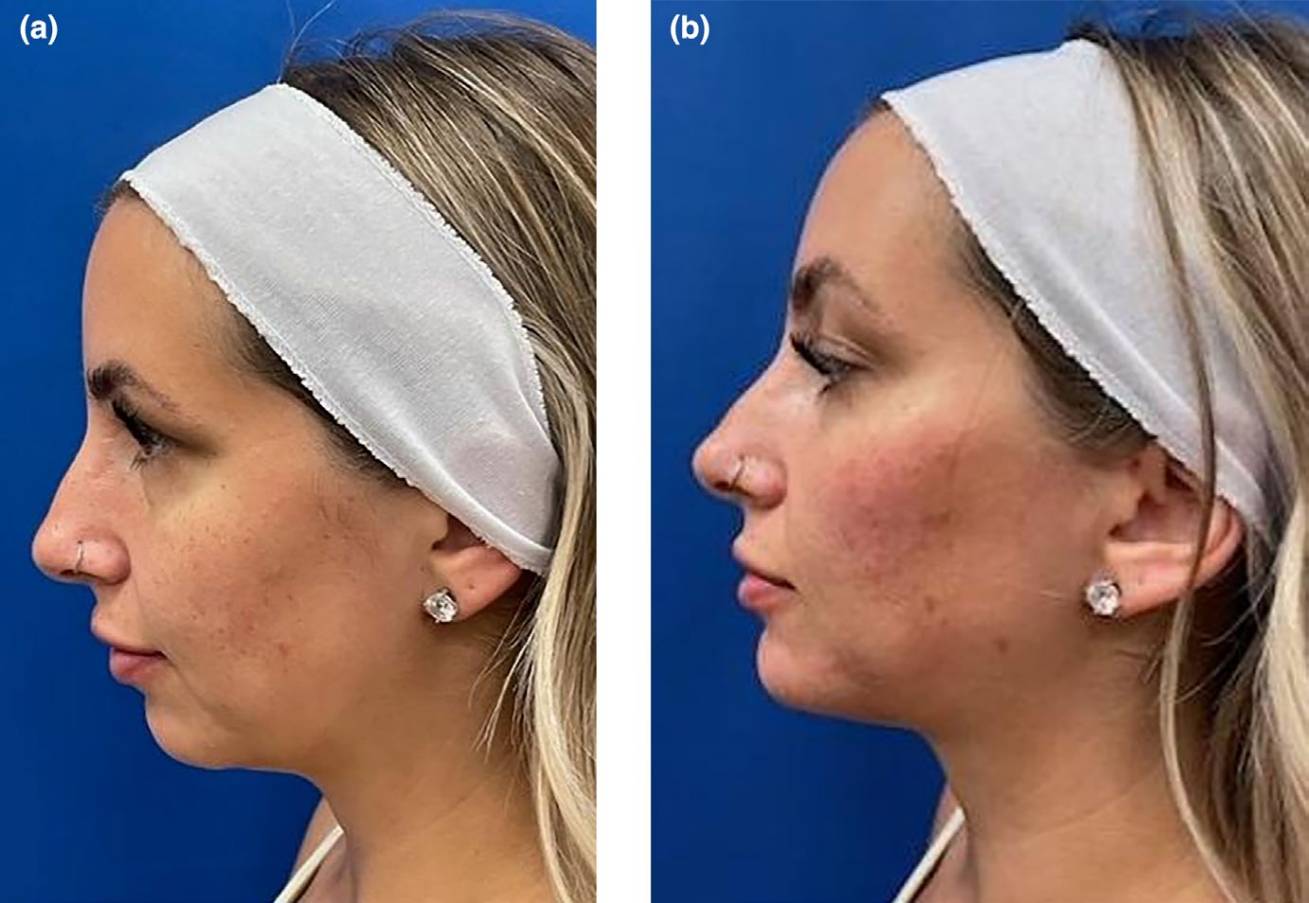
(a) Sagittal view of patient with microgenia and poor chin projection. (b) Same patient after 4 mL of high G' HA filler delivered in the subcutaneous plane to lateral cheeks and central chin. An additional 2 mL of the same filler was injected in the chin deep on bone with needle in the area for ideal female chin width, which respects the vertical lines dropped down from her inner canthus and ala. The after unfortunately shows a chin up position in comparison to her pre injection photograph, but it is still evident her profile was transformed from a convex to a straight profile.

This view is paramount as patients view themselves straight ahead in a mirror, but the world and social media often views them from the side or obliquely. A seasoned injector will also assess a patient from several views, including from above (bird's eye) and below (worm's eye) to restore natural facial curves. Taking a cell phone picture from the sagittal and oblique views will enlighten the patient as the benefits of facial shaping to improve their facial curves and profile. There are three main types of facial profiles: straight, convex, and concave (Figure 9). While straight is the most desirable, a convex profile will benefit from chin augmentation with a robust filler to create the desired straight profile (Figure 10).

There are several methods to inspect the sagittal view. One is Rickett's line that involves a line from the nasal tip to the chin. The upper lip should lie 4 mm behind this line while the lower 2 mm [8]. The benefit of this measurement is it demonstrates the importance of the delicate relationship between the nose and chin, and the effects they have on facial balance. More specifically, a recessed chin can cause the nose to appear more over projected, and an adequate chin gives the nose better balance. It can be challenging to determine 4 and 2 mm on a side view, so Reidel's (and Steiner's) line are other, simpler ways to assess chin prominence [8, 9]. A line is dropped from the upper to the lower lip, and this plane should be in line with the chin. The measurement is easy to perform with a cotton tip applicator and is based on the knowledge that the upper lip should project about 1–2 mm beyond the lower lip.

**FIGURE 11:**
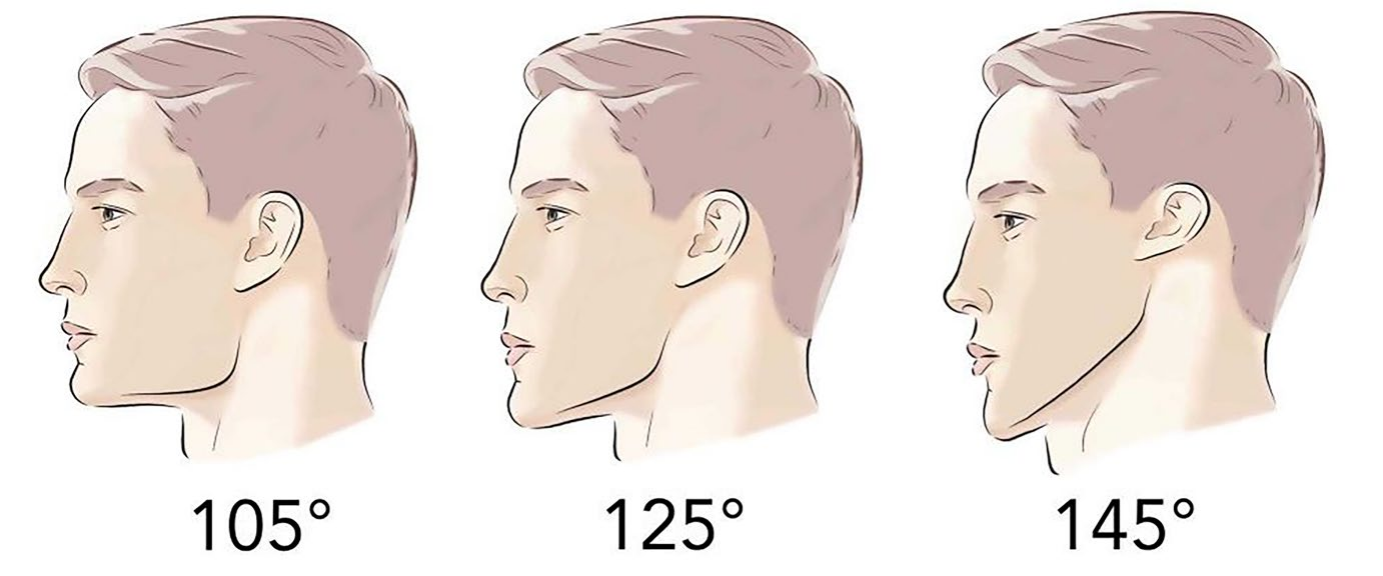
Gonial Angle: 127° is typically considered a desirable gonial angle. Men typically have sharper, more right‐angle contour than women. A larger, open angle over 130° is associated with a longer face and chin recession.

Looking at the patient's dental occlusion should be examined to provide clues as a class 2 malocclusion is associated with an overbite and a recessed chin. Also understanding the gonial angle is important as an optimal female angle is around 127° (Figure 11). Often, an open gonial angle of more than 130° is associated with a long face and chin retrusion.

### 1.3 E: External for Skin Envelop

The skin is the canvas of the face, and aging changes can be apparent with brown spots, hyperpigmentation, telangiectasia, lines, wrinkles, and deep folds along with actinic changes. There are a variety of options for improving and maintaining skin appearance. These modalities range from topical medical grade skin care and sunscreen to procedures such as microdermabrasion, chemical peels, and laser skin resurfacing. Recently, an injectable HA product specifically formulated for increasing skin hydration has become available in the United States [10].

### 1.4 S: Symmetry

**FIGURE 12:**
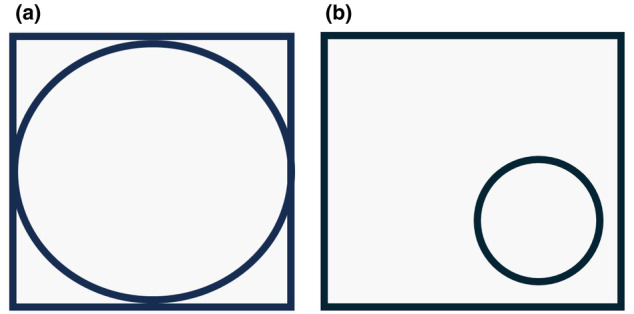
(a) When asked to draw a square and then a circle in the square, most people will try to fill the square with a circle that touched the square. (b) In fact, when asked to draw a circle in a box, very few people will draw a small, off‐center circle in the square. This is due to our innate belief that symmetry is beauty.

Symmetry has long been associated with beauty and is innately programmed in us. For instance, when asked to draw a circle and a square, a vast majority of people will try to fill the square evenly with a circle in an innate attempt to adhere to the underlying desire to achieve symmetry (Figure 12). Most people display a degree of facial imbalance or asymmetry, and this stems from our deep anatomic structures. The topographic appearance we witness on the surface is explained by the facial skeleton and boney foundation, deep and superficial fat pads, muscles of mastication, and skin quality. Perfect symmetry is associated with beauty, but perfect symmetry is exceedingly rare and illusive. In fact, many attractive people have facial asymmetry as witnessed when performing a simple split face view of the two right halves compared with a patient's two left halves. This view will invariably uncover a side with a higher eyebrow, bigger eye, and higher cheek, which is associated with our “good side.” This has been described as our baby and adult sides of our face respectively and stems from the differences in the skeletal foundation as being the origin of the asymmetry [11]. It is not actually important to correct all asymmetries as some features are often appealing and even considered attractive or distinctive. It is important, however, to identify and point out asymmetry prior to a cosmetic treatment to avoid an undesirable result.

### 1.5 S: Shadows

**FIGURE 13:**
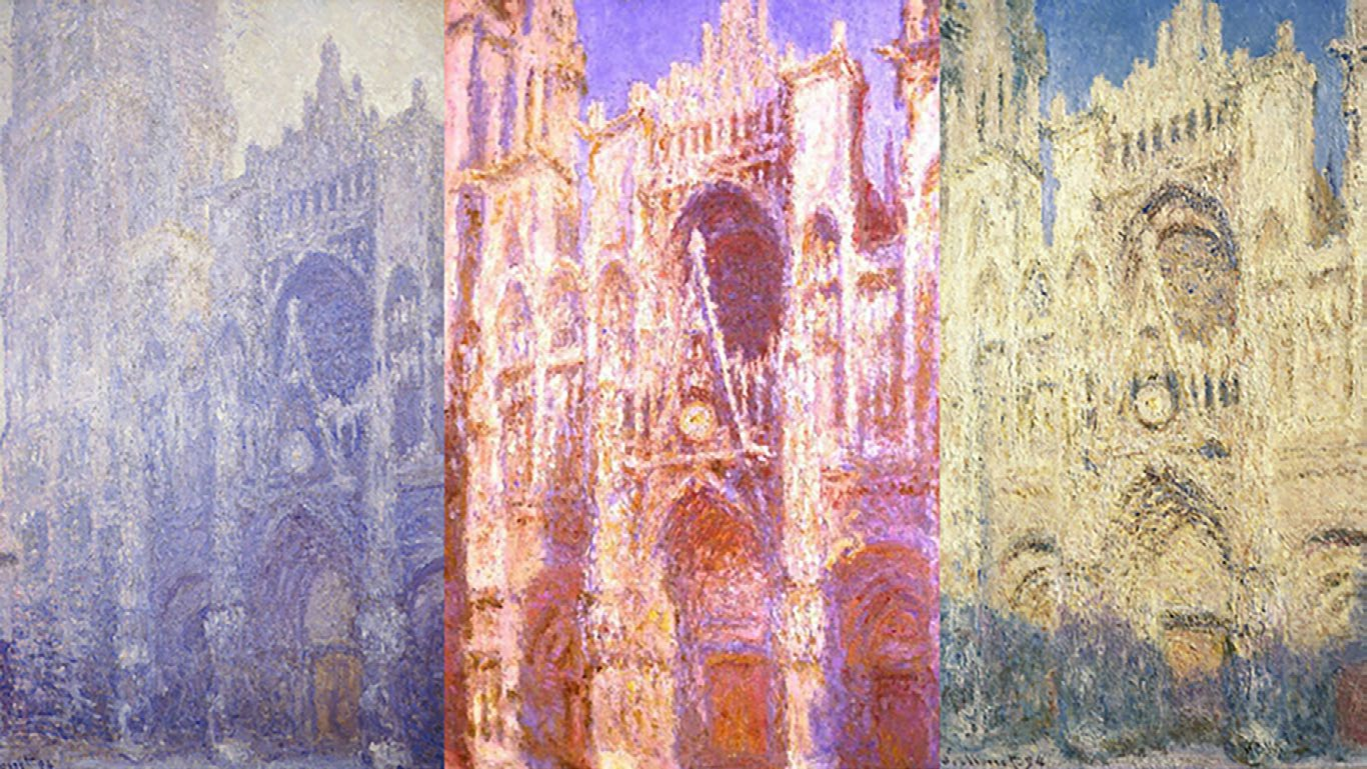
Monet's painting of the Rouen Cathedral painted in different lightening demonstrates the power of light and shadows on the appearance of an object or face.

The reflection of light on a face can produce vastly different appearances of the same image. For example, the image of Monet's painting of the Rouen Cathedral appears quite different in various shades of color and lighting (Figure 13). The same is true of our face and the manner it reflects light. Our face is made up of curves with convexities and concavities, referred to as ogee curves. This concept stems from an architectural reference of a convexity that transcends smoothly into a concavity, and facelift surgeons in the mid 1990s recognized the importance of ogee curves in delivering natural facelift results [12, 13]. The understanding that facial aging was partially caused by deflation in addition to descent lead to the concept that adding volume to a depleted, aged cheek was critical in restoring a youthful appearance such that skin is redraped over a convex surface rather than a flat cheek [14]. Multiple convexities exist in various areas of the face but are particularly present in the smooth round upper cheek that gently slopes into the concavity of the submalar area, and the convex forehead curve that descends into the concavity of the lateral orbit. This produces the noticeable double ogee curve of a facial profile and stems from the strong boney buttresses of the face around the superior and inferior orbit. Both these convexities are protected by deep fat pads, the ROOF and SOOF, and are responsible for protecting our brain and face, respectively. The recognition of natural facial curves stems from awareness that attractive facial shadows from concavities that trap light are just as important as convexities that reflect light in restoring balanced facial features. These concave areas, such as the submalar region, are critical to preserve in achieving beautiful cosmetic results. Failure to recognize these shadows can lead to an obliteration of these natural facial curves and result in an unnatural, round “pillow” face [15, 16].

## 2 Discussion

A critical eye and plan prior to treatment is key to delivering aesthetic outcomes. Facial assessment for filler and neurotoxin can be challenging, and the A.S.S.E.S.S. acronym provides an easy‐to‐follow method to address many key elements of facial assessment. By following the six steps outlined, the injector can perform a logical and reproducible assessment that can translate into delivering consistent filler and neurotoxin outcomes in clinical practice (Table 1). Others have recommended sound methods to analyze the face prior to filler injections or facial surgery, but they are more complex [17, 18, 19, 20, 21, 22, 23, 24, 25]. The beauty of using the word “assess” lies in its easy to remember acronym and simplicity of use. The A.S.S.E.S.S. method starts with animation as patients live in a dynamic state and injectors are encouraged to view the patient in movement to create the most natural results. Dynamic movement alters the facial structure and is critical in assessing the need for filler [16, 26]. Smiling raises the cheek soft tissues, and it is important not to overinflate the midface and create a sunken eye appearance. Animation also guides the injector as to neurotoxin needs and placement. The next two aspects of frontal facial shape and side view address the foundational components of the face and follow animation step. The filling of cheeks and chin creates a structural improvement in the face that can restore volume to deficient areas. By rebuilding loss volume from bone resorption and fat resorption, the overlying facial foundation is strengthened and restored to youthful proportions. These areas of the cheeks and chin can affect contiguous facial areas, such that filling the cheeks can create less of a need to fill the nearby nasolabial folds or tear troughs. A robust high G' filler is recommended when building structure in the midface and chin [27]. The external skin surface is often overlooked but is especially important for comprehensive facial rejuvenation. Often, skin care in addition to peels and sunblock should be considered. Analyzing facial symmetry is the fifth step and is critical in pointing out the differences between the right and left sides of the face. The two halves of the face should be considered siblings and not identical twins, and often, it is difficult to correct all asymmetries. Lastly, facial shadows should be respected as the face exists as a delicate balance of natural convexities and concavities. Once the facial foundation is treated with robust fillers, the fine lines that remain can be addressed by injecting a lower G' HA filler in areas such as the nasolabial folds and marionette lines. While the A.S.S.E.S.S. acronym provides a simple method for assessing the face for facial fillers, it is not a comprehensive approach to facial assessment and a more individualized, advanced assessment may be required for more complex scenarios. The AS.S.E.S.S. method does provide a solid basis for most patients in providing key information in facial analysis prior to filler and neurotoxin injections. Future directions for facial analysis potentially utilize AI or digital programs that can capture a patient's facial image, analyze facial form, and provide guidance in treatment paradigms.

**TABLE 1:** How to use the A.S.S.E.S.S. in six simple steps.

Step 1	A: Animate	Have patient smile and frown to assess facial nerve function	Use neuromodulator to soften dynamic lines and smooth wrinkles
Step 2	S: Facial shape	Determine patients facial shape on front view	Add structural high G' filler to temples, lateral cheeks, and chin to improve frontal facial shape as needed Consider neurotoxin to masseters to reduce fullness in a square face
Step 3	S: Side view	Determine patient's profile (straight, convex, and concave)	Add high G' HA filler to chin and jawline to improve a convex profile if present. Consider HA filler to pyriform and medial cheeks if deficiency exists
Step 4	E: External	Examine skin for wrinkles, dyschromias, and skin health	Recommend skin care with sunblock, medical grade skin care, and peels to improve skin appearance
Step 5	S: Symmetry	Determine which facial side is higher and point out the good side to the patient	Make patient aware of preexisting facial asymmetries and explain some of these differences between facial sides cannot be corrected with filler
Step 6	S: Shadows	Look for lines and wrinkles to fill	Fill lines such as nasolabial and marionette lines with HA filler after structural fillers placed in cheeks and chin

## 3 Conclusion

The A.S.S.S.E.S.S. acronym is easy to remember and a predictable method to assess a patient's face prior to filler and neurotoxin injections. Following the sequence of the six simple steps, outlined allows the injector to capture key elements of facial analysis prior to injectable procedures. The A.S.S.E.S.S. approach provides injectors with a consistent approach to deliver beautiful results.

## Author Contributions

The individual contributions of each author are as follows: This was a collaborative effort and all authors, J.P.F., S.B., J.W., R.F., J.D.T., and W.L., performed the research and concept. All authors (J.P.F., S.B., J.W., R.F., J.D.T., and W.L.) have read and approved the final manuscript. J.P.F. and R.F. performed the research and designed the research study. J.W., S.B., W.L., and J.D.T. contributed essential reagents or tools, pictures, and artwork. J.P.F., S.B., J.W., and W.L. analyzed the data. J.P.F. and R.F. authored the paper.

## Conflicts of Interest

John P. Fezza: Consultant for Allergan, Evolus, Revance, RVL, Arya Medical. Sheila Barbarino, MD: Speaker Galderma, Merz, SkinCeuticals, Miracu Threads. Julie Woodward: Consultant for Allergan, Galderma, Merz Aesthetics, Prollenium, SkinCeuticals, Horizon. Wendy Lee: Consultant for Allergan, Galderma, Revance, Evolus, Tarsus, RVL, Horizon, Novabay, RoC. Jonathan D. Tijerina, MD, MA: No disclosures. Reed Fezza: No disclosures.

## Data Availability

The data that support the findings of this study are available on request from the corresponding author. The data are not publicly available due to privacy or ethical restrictions.

## References

[1] L. G. Farkas and J. C. Kolar , “Anthropometries and Art in the Aesthetics of Women's Faces,” Clinics in Plastic Surgery 14, no. 4 (1987): 599–616. 3652607

[2] A. Swift and K. Remington , “BeautiPHIcation™: A Global Approach to Facial Beauty,” Clinics in Plastic Surgery 38, no. 3 (2011): 347–377. 21824535 10.1016/j.cps.2011.03.012

[3] T. Akinbiyi , S. Othman , O. Familusi , C. Calvert , E. B. Card , and I. Percec , “Better Results in Facial Rejuvenation With Fillers,” Plastic and Reconstructive Surgery. Global Open 8, no. 10 (2020): e2763. 33173655 10.1097/GOX.0000000000002763 PMC7647625

[4] A. Henderson , “Focusing on Chin Augmentation: Treating the Lower Face Using Dermal Filler,” Journal of Aesthetic Nursing 9 (2020): 191–194.

[5] S. Cotofana , K. C. Koban , F. Konstantin , et al., “The Surface‐Volume Coefficient of the Superficial and Deep Facial Fat Compartments: A Cadaveric Three‐Dimensional Volumetric Analysis,” Plastic and Reconstructive Surgery 143, no. 6 (2019): 1605–1613, 10.1097/PRS.0000000000005524. 30907804

[6] B. Mendelson and C. Wong , “Anatomy of the Aging Face,” In Aesthetic Surgery of the Face 6 Anatomy of the Aging Face sec. 1 Elsevier, 2012: 14124211.

[7] G. Casabona , K. Frank , K. C. Koban , et al., “Lifting vs. Volumizing—The Difference in Facial Minimally Invasive Procedures When Respecting the Line of Ligaments,” Journal of Cosmetic Dermatology 18, no. 5 (2019): 303–311, 10.1111/jocd.13245. 31402563

[8] P. H. Buschang , K. Fretty , and P. M. Campbell , “Can Commonly Used Profile Planes Be Used to Evaluate Changes in Lower Lip Position?,” Angle Orthodontist 81, no. 4 (2011): 557–563, 10.2319/081710-483. 21299383 PMC8919757

[9] B. Guyuron , “MOC‐PS(SM) CME Article: Genioplasty,” Plastic and Reconstructive Surgery 121, no. 4 (2008): 1–7. 10.1097/01.prs.0000305931.98111.c3 18379385

[10] M. Safa , A. Natalizio , and C. K. Hee , “A Prospective, Open‐Label Study to Evaluate the Impact of VYC‐12L Injection on Skin Quality Attributes in Healthy Volunteers,” Clinical, Cosmetic and Investigational Dermatology 15 (2022): 411–426. 35300433 10.2147/CCID.S352007 PMC8921677

[11] M. Divaris , E. Sabri , and S. Ohana , “Mirror Face Lift: Concept, Description, and Evaluation 1 Year Postoperatively,” Plastic and Reconstructive Surgery. Global Open 8, no. 3 (2020): e269. 10.1097/GOX.0000000000002697 PMC7253247 32537353

[12] J. W. Little , “Volumetric Perceptions in Midfacial Aging With Altered Priorities for Rejuvenation,” Plastic and Reconstructive Surgery 105 (2000): 252–266. 10626998 10.1097/00006534-200001000-00043

[13] O. M. Ramirez , “Fourth Generation Subperiosteal Approach to the Midface: The Tridimensional Functional Cheek Lift,” Aesthetic Surgery Journal 18, no. 2 (1998): 133–135, 10.1016/S1090-820X(98)80012-5. 19328121

[14] V. Lambros , “Observations on Periorbital and Midface Aging,” Plastic and Reconstructive Surgery 120, no. 5 (2007): 1367–1376. 17898614 10.1097/01.prs.0000279348.09156.c3

[15] T. S. Lim , R. Wanitphakdeedecha , and K. H. Yi , “Exploring Facial Overfilled Syndrome From the Perspective of Anatomy and the Mismatched Delivery of Fillers,” Journal of Cosmetic Dermatology 23, no. 6 (2024): 1964–1968. 38369859 10.1111/jocd.16244

[16] L. Schelke , S. Harris , H. Cartier , et al., “Treating Facial Overfilled Syndrome With Impaired Facial Expression‐Presenting Clinical Experience With Ultrasound Imaging,” Journal of Cosmetic Dermatology 22, no. 12 (2023): 3252–3260. 37772766 10.1111/jocd.16013

[17] M. de Maio , A. Swift , M. Signorini , and S. Fagien , “Aesthetic Leaders in Facial Aesthetics Consensus Committee. Facial Assessment and Injection Guide for Botulinum Toxin and Injectable Hyaluronic Acid Fillers: Focus on the Upper Face,” Plastic and Reconstructive Surgery 140, no. 2 (2017): 265e–276e. 10.1097/PRS.0000000000003544 28746271

[18] M. de Maio , K. DeBoulle , A. Braz , and R. J. Rohrich , “Alliance for the Future of Aesthetics Consensus Committee. Facial Assessment and Injection Guide for Botulinum Toxin and Injectable Hyaluronic Acid Fillers: Focus on the Midface,” Plastic and Reconstructive Surgery 140, no. 4 (2017): 540e–550e. 10.1097/PRS.0000000000003716 28953721

[19] M. de Maio , W. T. L. Wu , G. J. Goodman , and G. Monheit , “Alliance for the Future of Aesthetics Consensus Committee. Facial Assessment and Injection Guide for Botulinum Toxin and Injectable Hyaluronic Acid Fillers: Focus on the Lower Face,” Plastic and Reconstructive Surgery 140, no. 3 (2017): 393e–404e. 10.1097/PRS.0000000000003646 28841604

[20] A. Braz and C. C. P. Eduardo , “Reshaping the Lower Face Using Injectable Fillers,” Indian Journal of Plastic Surgery 53, no. 2 (2020): 207–218. 32884187 10.1055/s-0040-1716185 PMC7458843

[21] A. Braz and C. C. P. Eduardo , “The Facial Shapes in Planning the Treatment With Injectable Fillers,” Indian Journal of Plastic Surgery 53, no. 2 (2020): 230–243. 32884189 10.1055/s-0040-1715554 PMC7458834

[22] L. Farolch‐Prats and C. Nome‐Chamorro , “Facial Contouring by Using Dermal Fillers and Botulinum Toxin A: A Practical Approach,” Aesthetic Plastic Surgery 43, no. 3 (2019): 793–802. 30953112 10.1007/s00266-019-01361-1 PMC6522458

[23] A. J. Wilson , A. J. Taglienti , C. S. Chang , D. W. Low , and I. Percec , “Current Applications of Facial Volumization With Fillers,” Plastic and Reconstructive Surgery 137, no. 5 (2016): 872e–889e. 10.1097/PRS.0000000000002238 27119950

[24] J. Faryan and R. Rohrich , “The 10‐7 Facial Analysis Method for Face Lifting and Facial Rejuvenation,” Plastic and Reconstructive Surgery 154, no. 2 (2024): 275e–282e. 37220216 10.1097/PRS.0000000000010739

[25] R. Fitzgerald , M. Graivier , M. Kane , et al., “Facial Aesthetic Analysis,” Aesthetic Surgery Journal 30, no. 1_Supp (2010): 25S–27S. 20844297 10.1177/1090820X10373360

[26] I. Percec , V. Bertucci , N. Solish , T. Wagner , A. Nogueira , and J. Mashburn , “An Objective, Quantitative, Dynamic Assessment of Hyaluronic Acid Fillers That Adapt to Facial Movement,” Plastic and Reconstructive Surgery 145, no. 2 (2020): 295e–305e. 10.1097/PRS.0000000000006461 PMC7004449 31985621

[27] K. Beer , J. Kaufman‐Janette , D. Bank , et al., “Safe and Effective Chin Augmentation With the Hyaluronic Acid Injectable Filler, VYC‐20L,” Dermatologic Surgery 47, no. 1 (2021): 80–85. 33347003 10.1097/DSS.0000000000002795 PMC7752233

